# Neurofeedback-induced desynchronization of sensorimotor rhythm elicits pre-movement downregulation of intracortical inhibition that shortens simple reaction time in humans: A double-blind, sham-controlled randomized study

**DOI:** 10.1162/imag_a_00383

**Published:** 2024-12-11

**Authors:** Yoshihito Muraoka, Seitaro Iwama, Junichi Ushiba

**Affiliations:** School of Fundamental Science and Technology, Graduate School of Keio University, Kanagawa, Japan; Department of Biosciences and Informatics, Faculty of Science and Technology, Keio University, Kanagawa, Japan

**Keywords:** motor performance, transcranial magnetic stimulation (TMS), movement preparation, neurofeedback, sensorimotor rhythm (SMR)

## Abstract

Sensorimotor rhythm event-related desynchronization (SMR-ERD) is associated with the activities of cortical inhibitory circuits in the motor cortex. The self-regulation of SMR-ERD through neurofeedback training has demonstrated that successful SMR-ERD regulation improves motor performance. However, the training-induced changes in neural dynamics in the motor cortex underlying performance improvement remain unclear. Here, we hypothesized that SMR-neurofeedback based on motor imagery reduces cortical inhibitory activities during motor preparation, leading to shortened reaction time due to the repetitive recruitment of neural populations shared with motor imagery and movement preparation. To test this, we conducted a double-blind, sham-controlled study on 24 participants using neurofeedback training and pre- and post-training evaluation for simple reaction time tests and cortical inhibitory activity using short-interval intracortical inhibition (SICI). The results showed that veritable neurofeedback training effectively enhanced SMR-ERD in healthy male and female participants, accompanied by reduced simple reaction times and pre-movement SICI. Furthermore, SMR-ERD changes correlated with changes in pre-movement cortical disinhibition, and the disinhibition magnitude correlated with behavioral changes. These results suggest that SMR-neurofeedback modulates cortical inhibitory circuits during movement preparation, thereby enhancing motor performance.

## 1 Introduction

Neurofeedback is a technique that allows individuals to self-regulate brain activity through real-time feedback of neural signals, which may be presented visually, auditorily, or through other sensory modalities. Neurofeedback training (NFT), a form of self-regulation training, has demonstrated that induced sensorimotor plasticity improves human motor abilities (Onagawa et al., 2023;Ros et al., 2010;Sitaram et al., 2017). NFT approaches to manipulate endogenous neural activities in a non-invasive manner have been applied to a variety of motor tasks, such as simple or choice reaction tasks (Al-Wasity et al., 2021;Chiew et al., 2012;Doppelmayr & Weber, 2011;Egner & Gruzelier, 2004;He et al., 2020), force and posture control (Blefari et al., 2015;Maszczyk et al., 2018;Naros et al., 2016), as well as motor sequence production (Marins et al., 2019;Reiner et al., 2018;Rozengurt et al., 2016). Recent meta-analyses have revealed that NFT-induced activity changes in targeted regions, especially sensorimotor cortices (SMC), can augment human motor performance (Mirifar et al., 2017;Onagawa et al., 2023;Xiang et al., 2018).

A characteristic component of neural signals used in the NFT paradigm is the sensorimotor rhythm (SMR). SMR signals found in scalp electroencephalograms (EEG) are signatures of rhythmic SMC activities at 7–24 Hz, purportedly paced by the thalamo-cortical loop (Pfurtscheller & da Silva, 1999;Seeber et al., 2019). Evidenced by physiological properties consistent with those found in invasive and noninvasive recordings, such as task-related spectral power attenuation (Miller et al., 2007,^2010^;Naros et al., 2020), EEG-SMR reliably represents corticomotor excitability changes. The event-related desynchronization of SMR (SMR-ERD) during execution, imagery, and preparation of movement is associated with changes in the excitability of descending corticomotor pathways and disinhibition of intracortical inhibition (Hummel et al., 2002;Takemi et al., 2013,^2015^,^2018^). Collectively, inducing SMR-ERD via NFT would manipulate the underlying motor circuitries and associated motor performance.

NFT for SMR-ERD systematically improves motor performance, presumably by learning voluntary corticomotor excitability changes (Kraus et al., 2016,^2018^;Ros et al., 2010). However, the neurophysiological effects of NFT on motor cortical circuitries and how it leads to performance improvement remain unclear. If SMR-ERD NFT involves the neural resources shared with motor tasks, the neural population would become more responsive after training (Mihelj et al., 2021;Miller et al., 2010;Soekadar et al., 2015). Therefore, the contribution of NFT-induced neural plastic changes in SMC can be revealed by assessing the reorganization of SMC circuitries using motor-evoked potential (MEP) magnitude derived from transcranial magnetic stimulation (TMS).

Here, we tested the hypothesis that NFT-induced SMC excitability changes modulate cortical inhibitory circuitries, assessed using the paired-pulse TMS protocol to measure short-latency intracortical inhibition (SICI) magnitude (Kujirai et al., 1993;Ziemann et al., 1996). To this end, we employed a simple reaction time task to measure behavioral performance improvement and MEP during the movement preparation period before and after the intervention (Coxon et al., 2006;Davey et al., 1998;Duclos et al., 2008;Duque & Ivry, 2009;Heise et al., 2013;Hummel et al., 2009). The NFT-induced functional plasticity in the trained primary motor cortex (M1) was evaluated in a randomized, sham-controlled design (Ros et al., 2020;Sorger et al., 2019). Participants were randomly allocated to real or yoked-sham placebo groups and experienced a 40-minute SMR-ERD-NFT based on their real-time EEG signals, or those previously acquired from others, allowing us to highlight the role of closed-loop neurofeedback in inducing excitability changes in the target area.

## 2 Methods

### 2.1 Study design

The present study adopted a double-blind, randomized controlled design, and the experiment was conducted in accordance with the CONSORT statement and CRED-nf checklist (Ros et al., 2020;Schulz et al., 2010). Participants, the experimenter, and the data analyst were blinded to group allocation. The sample size was determined using power analysis, computed by G*Power software (version 3.1.9.6) for α = 0.05, 1-β = 0.8, and d = 0.60 (Faul et al., 2007). The effect size was determined based on the results of reaction time (RT) change in preliminary experiments. A total of 24 participants were required, which is comparable to other NFT studies (Hayashi et al., 2020;He et al., 2020).

### 2.2 Participants

Twenty-four volunteers, aged 18–28 (17 males, 7 females), were enrolled as planned. Twenty participants were right-handed according to the Edinburgh Handedness Inventory (Oldfield, 1971). All participants were naïve to the purpose of the experiment and the procedures, with no history of neurological disorders or other health concerns. The purpose and experimental procedure were explained to the participants before written informed consent was obtained. The study was approved by the local ethics committee of Keio University (IRB approved number: 2021-118) and performed in accordance with the Declaration of Helsinki.

Two participants were excluded due to excessive contraction of finger muscles during neurofeedback training, despite repetitive instruction. Consequently, 22 participants experienced real NFT (real-group, N = 11, age: 22.3 ± 2.5, 3 females, 0 left-handed) or sham NFT (sham-group, N = 11, age: 21.1 ± 2.1, 2 females, 3 left-handed).

### 2.3 Experiment protocol

**Fig. 1.**
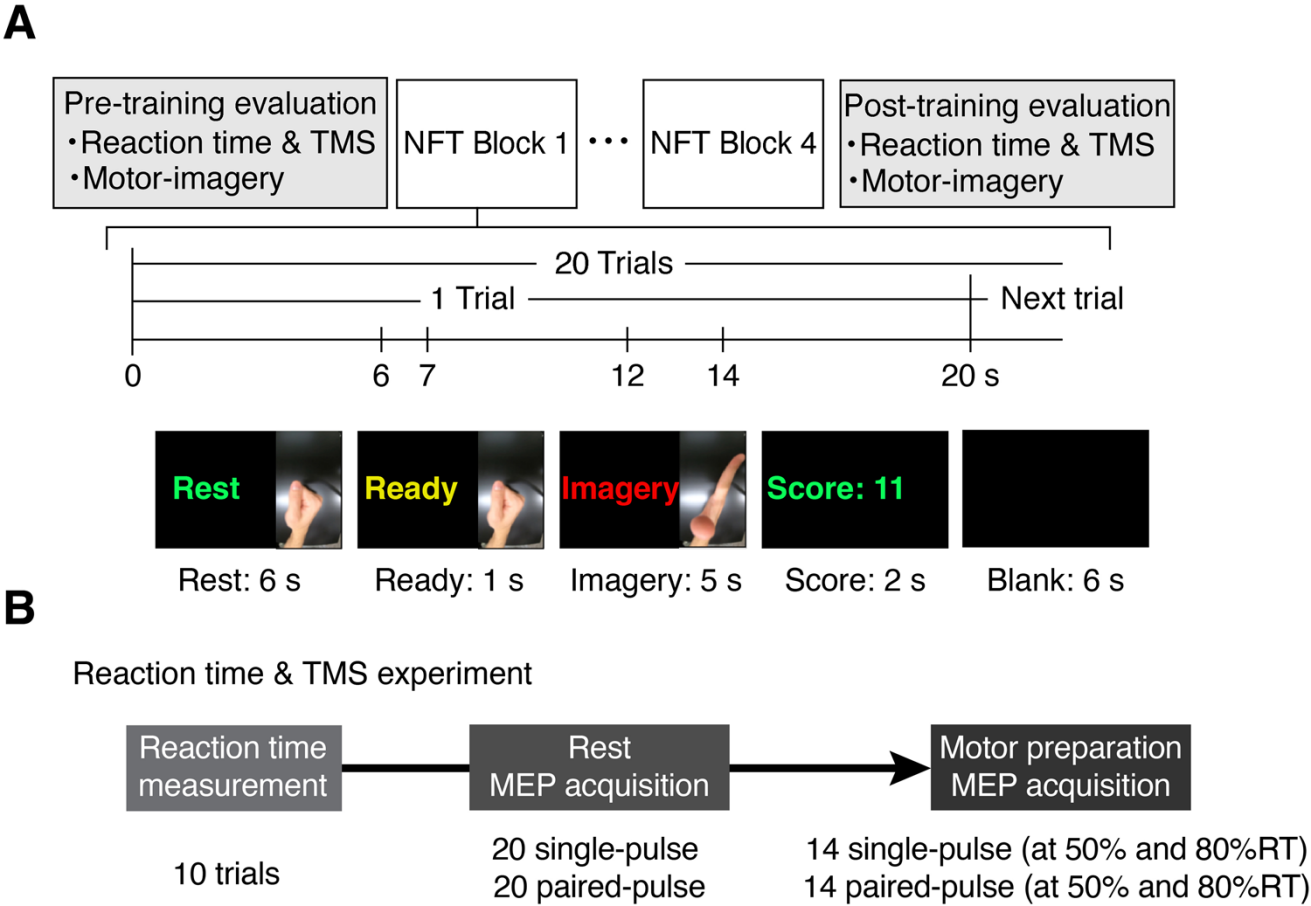
Experimental paradigm and EEG recording. (A) An overview of the experimental protocol. Participants performed four NFT (neurofeedback training) blocks and pre- and post-training evaluations. During NFT blocks, participants were instructed to control a hand image on the screen during the imagery epoch. (B) During pre- and post-training evaluation, participants underwent reaction time and TMS experiments. These included reaction time measurements, resting MEP acquisition, and motor preparation MEP acquisition.

Each participant experienced four NFT blocks interleaved with pre- and post-training evaluation blocks (Fig. 1A). Evaluation blocks comprised behavioral tests for reaction time and TMS measurements, as well as scalp EEG measurements during motor imagery. An NFT block comprised 20 trials. All experimental procedures were completed within 3 hours.

#### 2.3.1 Behavioral test

The behavioral test consisted of the simple reaction time task and TMS evaluations. During the test, surface electromyography (EMG) signals of the extensor digitorum communis (EDC) muscle were recorded to capture finger extension onset. The “Go” signal (visual cue) was generated every 6 ± 1 seconds, and participants were instructed to open their right hand as quickly as possible in response to the cue. RTs were defined as the time interval between the “Go” signal and the onset of EMG bursts. EMG onsets were defined as the time point when the data from the “Go” signal to 100 ms exceeded four times the standard deviation. All EMG data were visually inspected to reject trials where EMG potentiation was apparent before the “Go” cue. Participants performed 10 trials in the pre- and post-behavioral tests in the absence of TMS (Fig. 1B).

Single- and paired-pulse TMS was applied during resting and movement preparation conditions, with a protocol similar to previous studies (Heise et al., 2013;Hummel et al., 2009). In the resting condition, participants received a total of 20 trials of single-pulse and paired-pulse TMS in a pseudo-random order. In movement preparation conditions, participants performed the reaction time task while single- and paired-pulse TMS was applied in a pseudo-randomized order at 50% and 80% of pre-reaction time. Each timing and stimulation condition was repeated seven times in randomized order.

#### 2.3.2 Neurofeedback training

The protocol for NFT was based on previous studies employing SMR-ERD neurofeedback (Hayashi et al., 2020;Iwama et al., 2022;Kodama et al., 2023;Soekadar et al., 2015). NFT comprised four 7-minute blocks. Additionally, two evaluation blocks interleaved the NFT blocks. Participants had short breaks before starting a block. Each evaluation and training block consisted of 20 trials. Each trial began with a 6-second resting epoch, followed by a 1-second ready epoch, and completed by a 5-second motor imagery epoch. During this 12-second trial period, participants were asked to avoid moving, blinking, and swallowing. After each 12-second trial period, the screen showed the score of the trial for 2 seconds, followed by a 6-second interval. During the interval period, participants were allowed to move freely to avoid mental fatigue before the next trial started. EEG and EMG signals were recorded throughout neurofeedback training.

In the pre- and post-evaluation blocks, no visual feedback was provided to verify the learning effect of closed-loop NFT in the open-loop condition (Hayashi et al., 2020;Ruddy et al., 2018). The pre-training evaluation block measured baseline SMR-ERD magnitude, and EEG signals were used to calibrate NFT parameters for each participant to adjust the most responsive frequency (Kodama et al., 2023). Specifically, the target frequency was selected from the individual alpha frequency (IAF) by calculating the mean SMR-ERD magnitude with the most pronounced 3 Hz, including 8–13 Hz (Iwama & Ushiba, 2024). For offline analysis, the individual beta frequency (IBF) was defined as twice the median of IAF ± 2 Hz (Pichiorri et al., 2015).

**Fig. 2.**
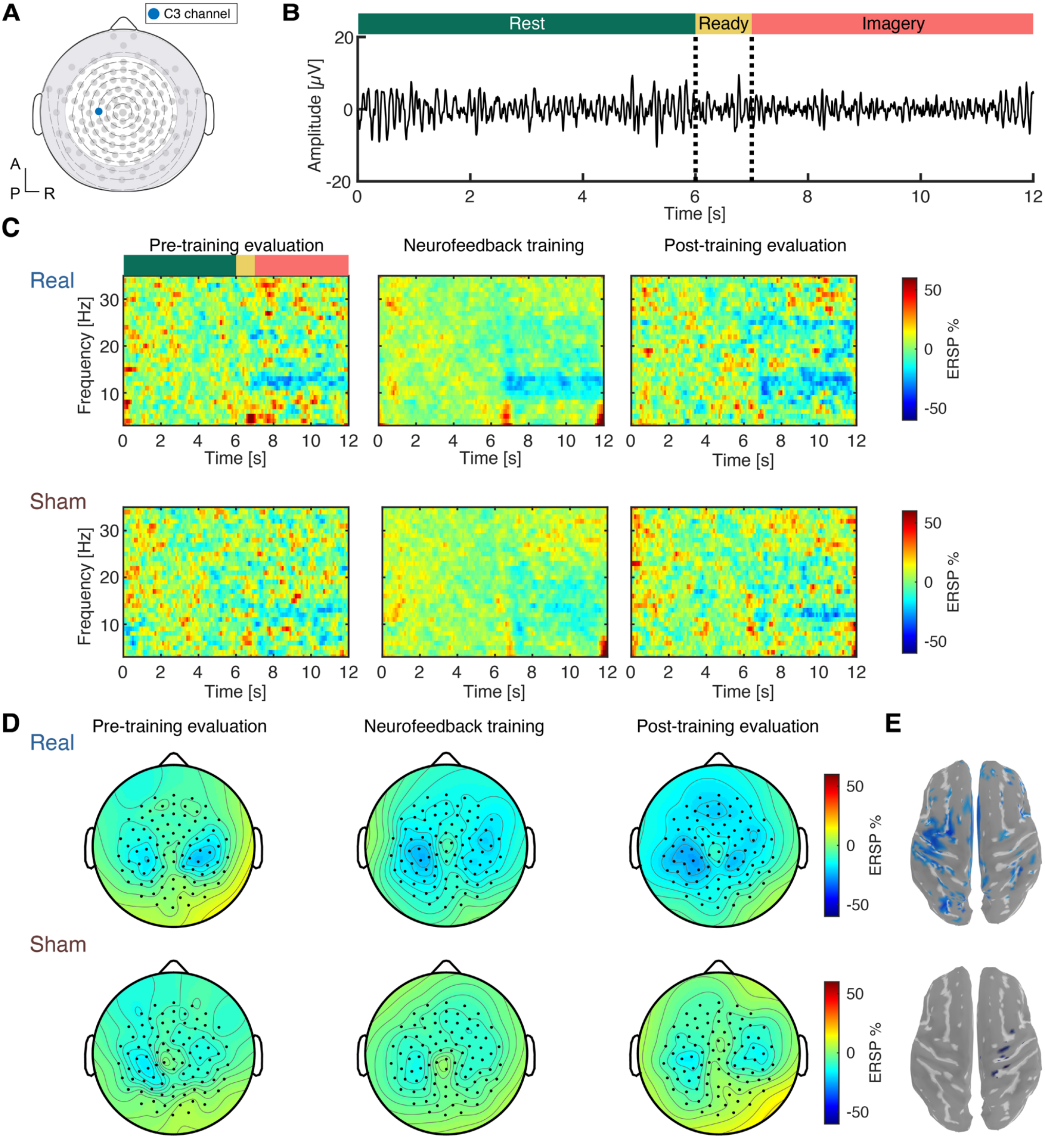
Time-frequency representations of sensorimotor rhythm. (A) Placement of EEG electrodes. Ninety-one electrodes outside the gray shades were used for EEG analysis. The blue electrode is the C3 channel in the international 10-20 system used for NFT. (B) Raw signals of sensorimotor rhythm derived from scalp EEG over the sensorimotor cortex (i.e., C3 channel) during a representative trial. Induced power attenuation during the motor imagery period is qualitatively observed. (C) Time-frequency representation of EEG signals at the C3 channel. Data averaged across participants and trials are shown. The upper panels indicate the real-group data during evaluation and training blocks, and the lower panels indicate the sham group data. The task-related power attenuation, represented as the blue band (negative event-related spectral perturbation: ERSP), is observed around 10 Hz. (D) Topographic representation of the ERSP magnitude during the imagery period. The individual data were averaged at their individual alpha frequencies. The negative ERSP values were bilaterally observed. (E) The source-level distribution of SMR-ERD magnitude at the post-training evaluation. The statistically significant areas were visualized by thresholding (*p*< 0.05, FDR-correction).

In the training blocks, participants received visual feedback based on SMR-ERD from the left sensorimotor cortex (Fig. 2A, C3 channel). The visual feedback was provided by hand images between flexion and extension (Ono et al., 2013;Pichiorri et al., 2015). The image was presented depending on the value of SMR-ERD (range: 0–100%). Participants were instructed to open the hand image during the motor imagery epoch.

The online SMR-ERD magnitude was calculated every 100 ms using the last 1-second data as follows. Raw EEG signals recorded from C3 were re-referenced by the six neighboring electrodes (Fig. 2A). This method is referred to as a large Laplacian and is known to improve the signal-to-noise ratio for sensorimotor mu rhythm (Mcfarland et al., 1997;Pfurtscheller & da Silva, 1999;Tsuchimoto et al., 2021). The re-referenced data underwent a 1–70 Hz fourth-order Butterworth bandpass filter and 50-Hz notch filter. Subsequently, the time series data were subjected to fast Fourier transform (FFT) with a 1-second sliding window, a Hanning window, and 90% overlap. The power spectrum density was calculated using FFT data. SMR-ERD was calculated using the following formula:

$$SMR–ERD=\frac{R\left(f\right)-A\left(f,t\right)}{R\left(f\right)}\times 100$$

whereAis the power spectrum density of the motor imagery epoch at timetand frequencyf, respectively.Ris the median power spectrum density of the resting epoch. For the sham feedback group, the visual feedback scores were calculated in the same way but the screen showed the scores of another participant (Kober et al., 2018;Kodama et al., 2023;Sorger et al., 2019).

#### 2.3.3 Experiment setup

Participants were seated on a comfortable chair with armrests in an unshielded experimental room. A display was set up about 1 meter in front of the chair to show visual feedback of hand images representing SMR-ERD scores and task instructions. All participants completed a pre-training behavioral test, neurofeedback training, and a post-training behavioral test (Fig. 1A).

### 2.4 Data acquisition

#### 2.4.1 Surface electromyography

EMG signals were recorded from the right-hand EDC muscles using bipolar Ag/AgCl electrodes. The cathode electrodes were placed over the muscle belly, and the anode was placed 20 mm distal from the cathode. Impedance for all channels was maintained below 20 kΩ throughout the experiment. Signals were band-pass filtered (10–500 Hz with a second-order Butterworth filter) and digitized at 10 kHz using a biosignal amplifier (Neuropack MEB-9200; Nihon Koden, Tokyo, Japan). The acquired signals were monitored throughout the experiment.

#### 2.4.2 Transcranial magnetic stimulation

TMS pulses were generated by two interconnected single-pulse magnetic stimulators with a 70 mm figure-of-eight coil (Magstim Bistim2; Magstim Co., UK). TMS was applied over the left M1. At this position, the coil was oriented approximately 45 degrees to the sagittal plane. The resting motor threshold (RMT) was defined as the lowest stimulator output eliciting an MEP in the relaxed EDC of >50 µV peak-to-peak in 5 out of 10 trials (Rossini et al., 1994). Single-pulse TMS was set at an intensity of 120% of the RMT. In paired-pulse TMS conditions, a subthreshold conditioning stimulus was set at 80% of the RMT and was delivered through the same magnetic coil at 2 ms prior to the suprathreshold test stimulus adjusted to 120% of the RMT (Takemi et al., 2013,^2018^;Vaalto et al., 2011). Three participants whose 120% RMT was higher than 100% maximum stimulator output were excluded from TMS evaluation. The stimulus intensity remained constant throughout the experiment for each participant.

#### 2.4.3 Electroencephalograms

EEG signals were recorded by the Electrical Geodesic system with a 128-channel HydroCel Geodesic Sensor Net (GES 400; Electrical Geodesics, Inc.) with a sampling rate of 1 kHz. Ground and reference electrodes were placed at CPz and Cz in the extended 10-20 system, respectively. Electrode impedance levels were kept below 40 kΩ throughout neurofeedback training.

### 2.5 Data analysis

All analyses of EMG and EEG signals were conducted using MATLAB 2020b (The MathWorks, Inc, Natick, MA, USA) and EEGLAB (Delorme & Makeig, 2004).

#### 2.5.1 Offline EEG analysis

EEG electrodes around the eyes and ears were excluded from further analyses to minimize the effect of artifacts related to non-neural activities. Consequently, EEG signals from the remaining 91 electrodes were used for further analyses (Fig. 2A). EEG signals were first band-pass filtered between 3–40 Hz and notch filtered at 50 Hz using a fourth-order zero-phase Butterworth filter. The filtered data were segmented between the resting epoch and MI epoch +1 second. Independent component analysis (ICA) was applied for artifact removal after bad trial rejection. The common average reference was used to enhance the signal-to-noise ratio and to extract localized activities (Mcfarland et al., 1997). Subsequently, a short-time Fourier transform (STFT) was applied with 1-second sliding windows, a Hanning window, and 90% overlap. Event-related spectral perturbation (ERSP) was calculated using the power spectrum density from STFT data to evaluate SMR-ERD. The median ERSP was calculated for each block and each participant. In the time-frequency map, the signal of C3 in EEG was used. To visualize the cortical source distribution modulated by the intervention, we used the sLORETA algorithm implemented in the Brainstorm toolbox (Pascual-Marqui, 2002;Tadel et al., 2011).

#### 2.5.2 MEP analysis

Peak-to-peak MEP amplitudes were calculated offline. The TMS data from five participants were discarded due to the absence of SICI (four participants) and equipment trouble (one participant). Recorded EMG signals were first band-pass filtered between 10–450 Hz and notch filtered at 50 Hz using a fourth-order Butterworth filter. The filtered data were segmented between 100 ms before and 150 ms after the stimulus. The resting-state and movement preparation state data were analyzed, respectively. For the resting condition data, MEP amplitudes were averaged for single-pulse and paired-pulse conditions. SICI was evaluated using the mean MEP amplitude provoked with a 2-ms interstimulus interval that showed smaller MEP. For the movement preparation condition data, MEP amplitudes were averaged for each TMS protocol (single-pulse and paired-pulse) and condition (pre-RT 50% and pre-RT 80%). To quantify SICI, the mean MEP amplitude by paired-pulse TMS was expressed as a percentage of the mean MEP amplitude by single-pulse TMS (Coxon et al., 2006;Hummel et al., 2009).

### 2.6 Statistics

#### 2.6.1 EEG

The ERSP magnitude during training and evaluation blocks, which represents SMR-ERD as a negative value, was analyzed using two-way mixed repeated-measures ANOVA (rmANOVA) models with Time (blocks) and Group (real or sham feedback) factors. If ANOVA yielded a significant*F*value, a post hoc analysis was performed using the Bonferroni test. The type I error was set to 0.05. Changes in the ERSP magnitude were assessed by a two-sample*t*-test. These analyses were conducted for ERSP in the alpha and beta-bands, respectively. The cortical source significantly manipulated during the imagery period was determined by*t*-test with the false-discovery rate correction.

#### 2.6.2 TMS

The single-pulse MEP and SICI magnitude were analyzed with a three-way rmANOVA with Condition (rest, RT 50%, and RT 80%), Time (pre- and post-training evaluation), and Group factors. In addition, the rmANOVA with Condition × Group was conducted to assess the difference in SICI between pre- and post-training evaluations. For the ANOVA model for SICI data, baseline MEP amplitude was included as a covariate since the magnitude of SICI was calculated based on the single-pulse MEP and is known to be influenced by the inter-individual difference in the responsiveness to single-pulse stimulation (Hummel et al., 2009;Takemi et al., 2018).

#### 2.6.3 Reaction time

Reaction time was analyzed using two-way mixed rmANOVA models with Time and Group factors. In addition, the standard mean difference was calculated for the integrated analysis of NFT results based on the recent meta-analysis (Onagawa et al., 2023).

#### 2.6.4 Correlation analysis

Between-participant correlations for changes in ERSP magnitude in the alpha- and beta-bands were analyzed using Pearson’s correlation test. The relationships among changes in ERSP, single-pulse MEP, SICI, and reaction time were evaluated with the partial correlation coefficient. Statistically significant (*p*< 0.05) pairs were reported.

## 3 Results

NFT, as well as behavioral tests did not lead to any adverse effects and all participants completed the experiment protocols.

### 3.1 Neurofeedback training guides task-related desynchronization of sensorimotor rhythm

We assessed the training-induced changes in SMR-ERD magnitude around the contralateral SMC during motor imagery of finger movement. During the training, SMR signals exhibited larger amplitude at the rest period while attenuated amplitude from ready to imagery period (Fig. 2B). The group time-frequency analysis from electrodes around SMC (C3 channel,Fig. 2C) showed task-related SMR-ERD (i.e., reduction of ERSP magnitude) during the imagery period in all groups and blocks. To evaluate the spatial distribution of spectral power change, topographic maps derived from whole-head scalp EEG were visualized (Fig. 2D). The sensor-space changes in the modulation depth of SMR-ERD magnitude were found in the verum group. This observation was corroborated by the source distribution shown inFigure 2E. The prominent SMR-ERD was observed around the bilateral SMC at IAF (12.0 ± 1.2 Hz).

**Fig. 3.**
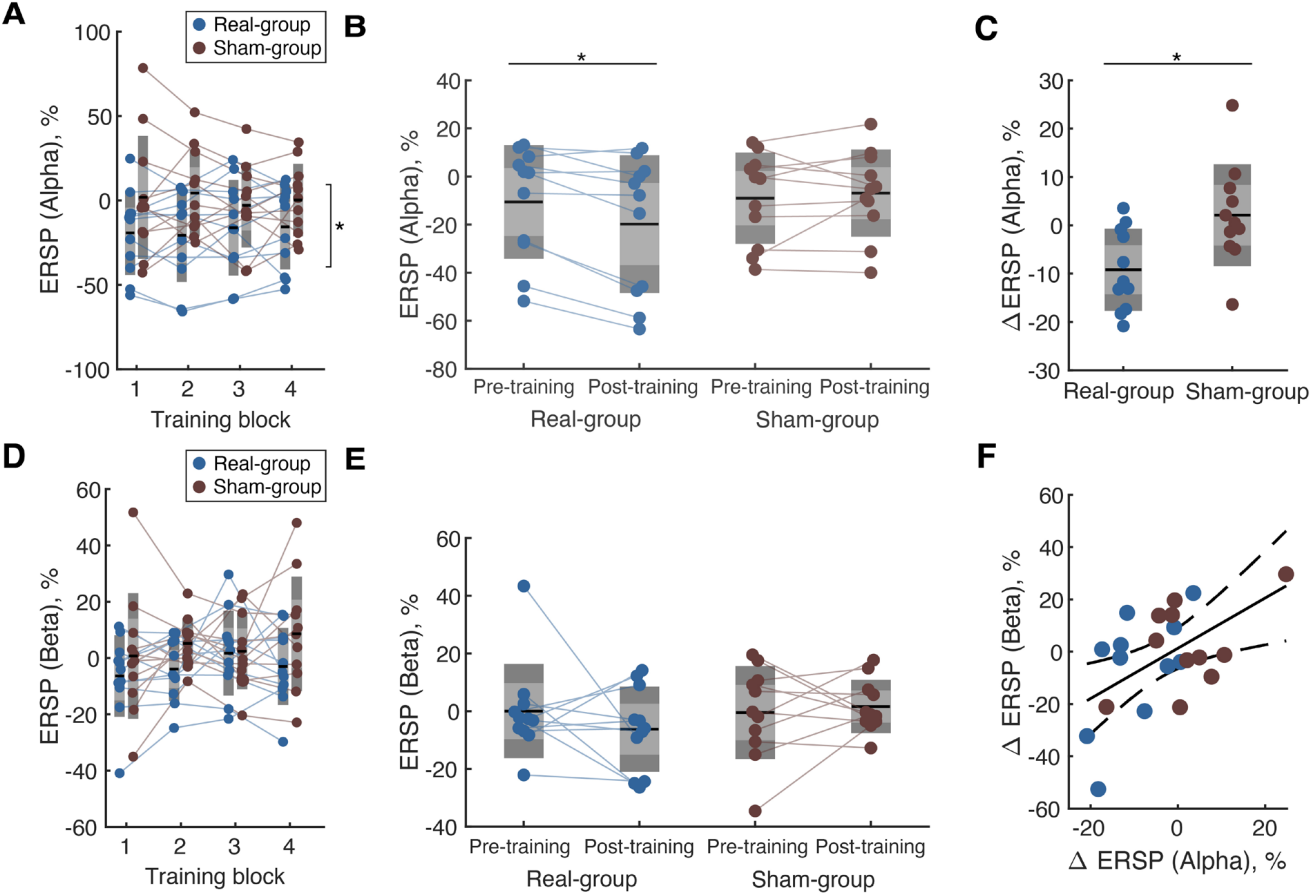
Training-induced spectral power changes in sensorimotor rhythm. Training-induced spectral power changes in sensorimotor rhythm. (A) EEG spectral power modulation during NFT. ERSP values at the individual alpha frequency were compared using a repeated-measures ANOVA. A significant main effect of group was found (*p< 0.05)*.*(B) ERSP values at evaluation blocks. A repeated-measures ANOVA revealed a significant interaction of Time and Group effects. Post-hoc analysis indicated a significant reduction in ERSP magnitude in the real group (*p< 0.05). (C) Between-group comparison of changes in ERSP values at evaluation blocks. A two-sample*t*-test revealed significant differences in the changes in ERSP values. (D) EEG spectral power modulation during NFT. ERSP values at the individual beta frequency were compared using a repeated-measures ANOVA. (E) ERSP values at evaluation blocks. (F) Relationship between changes in the ERSP magnitude at the alpha and beta bands.

The induced spectral power changes during NFT blocks were analyzed using SMR-ERD magnitude from the C3 channel. SMR-ERD magnitude at IAF revealed a significant main effect of the group (two-way mixed rmANOVA,*F*= 8.57,*p*= 0.009,Fig. 3A), and post-hoc test indicated that ERSP values were significantly smaller in the real-group (two-sample*t*-test,*t*= 2.93,*d*= 1.0), suggesting that stronger SMR-ERD was induced during training blocks in the real-group. For data during evaluation blocks, a significant Time × Group interaction was found (two-way mixed rmANOVA,*F*= 7.85,*p*= 0.01,Fig. 3B) and post-hoc analysis revealed that SMR-ERD at IAF was significantly enhanced in the real feedback group (Real:*t*= 3.23,*p*= 0.004,*d*= 0.41, Sham:*t*= -0.73,*p*= 0.91,*d*= 0.01). Consequently, the difference from the pre-training to the post-training block in the ERSP magnitude at IAF was significantly smaller in the real-group compared to the sham (Fig. 3C,*t*= -2.80,*p*= 0.01,*d*= 0.49; two-sample*t*-test).

Since it is known that SMR-ERD also occurs in the beta frequency during motor imagery (Pfurtscheller & da Silva, 1999), we also assessed beta-band SMR-ERD. We did not find a significant effect in the data during training and evaluation blocks (two-way mixed rmANOVA,Fig. 3D, E), and changes in ERSP magnitude at the beta-band were significantly correlated with those in the alpha-band (Pearson’s correlation test,*r*= 0.54,*p*= 0.009;Fig. 3F).

### 3.2 Neurofeedback training reduces simple reaction time

**Fig. 4.**
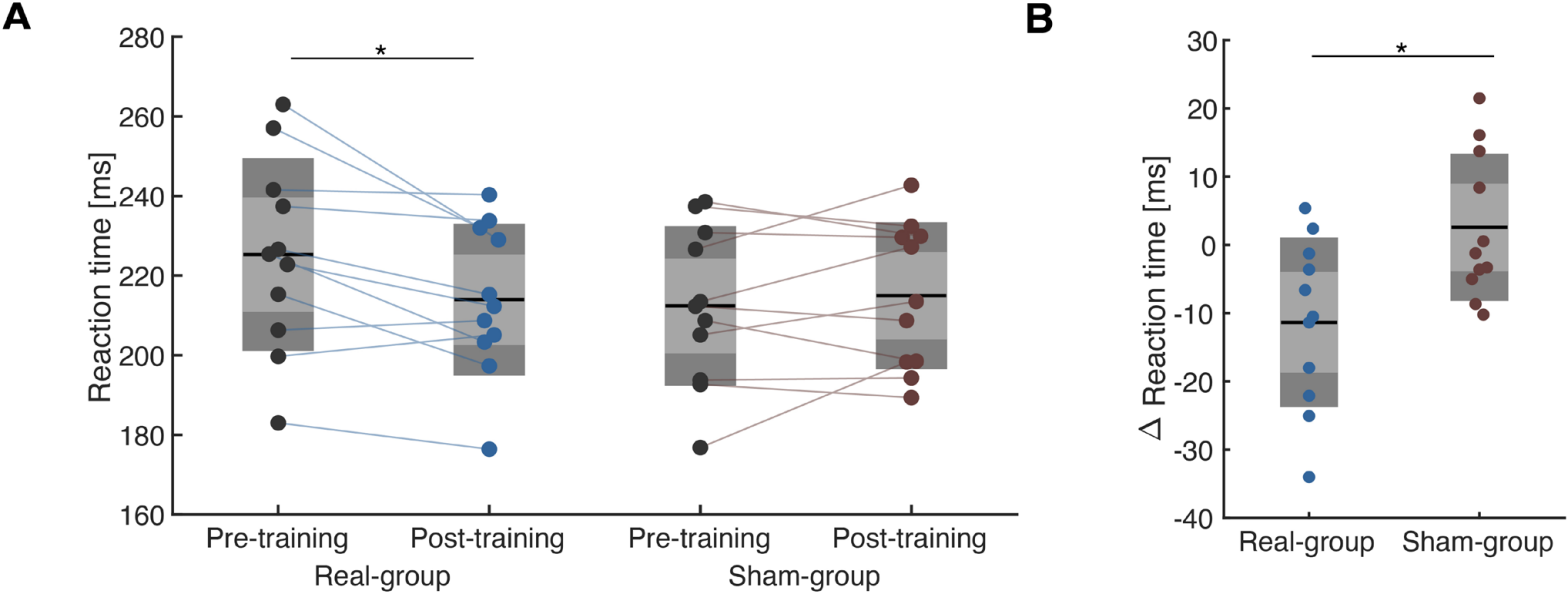
Neurofeedback-induced reaction time change. (A) Reaction time (RT) at evaluation blocks. A repeated-measures ANOVA revealed a significant interaction of Time and Group effects. Post-hoc analysis indicated a significant reduction in RT in the real group (*p< 0.05). (B) Between-group comparison of changes in RT values at evaluation blocks. A two-sample*t*-test revealed significant differences in the changes in RT. A two-sample*t*-test revealed significant differences in the changes in RT (*p< 0.05).

Simple reaction time was measured before and after NFT to determine if the trained SMR-ERD magnitude could further modulate voluntary corticomotor output (Fig. 4). The RT analysis using a two-way rmANOVA revealed significant interaction of Time × Group (*F*= 8.02,*p*= 0.01,Fig. 4A), and post-hoc*t*-test revealed that reaction time was significantly reduced in the real feedback group (Real:*t*= 3.05,*p*= 0.01,*d*= 0.58, Sham:*t*= -0.80,*p*= 0.44; paired*t*-test). The baseline RT performance did not indicate a significant difference (two-sample*t*-test,*t*= 1.38,*p*= 0.18). The reaction time became approximately 10 ms faster in the real feedback group before and after neurofeedback training (*t*= -2.83,*p*= 0.01,*d*= 1.20; unpaired*t*-test,Fig. 4B).

### 3.3 Stronger disinhibition of intracortical inhibition was induced during movement preparation

**Fig. 5.**
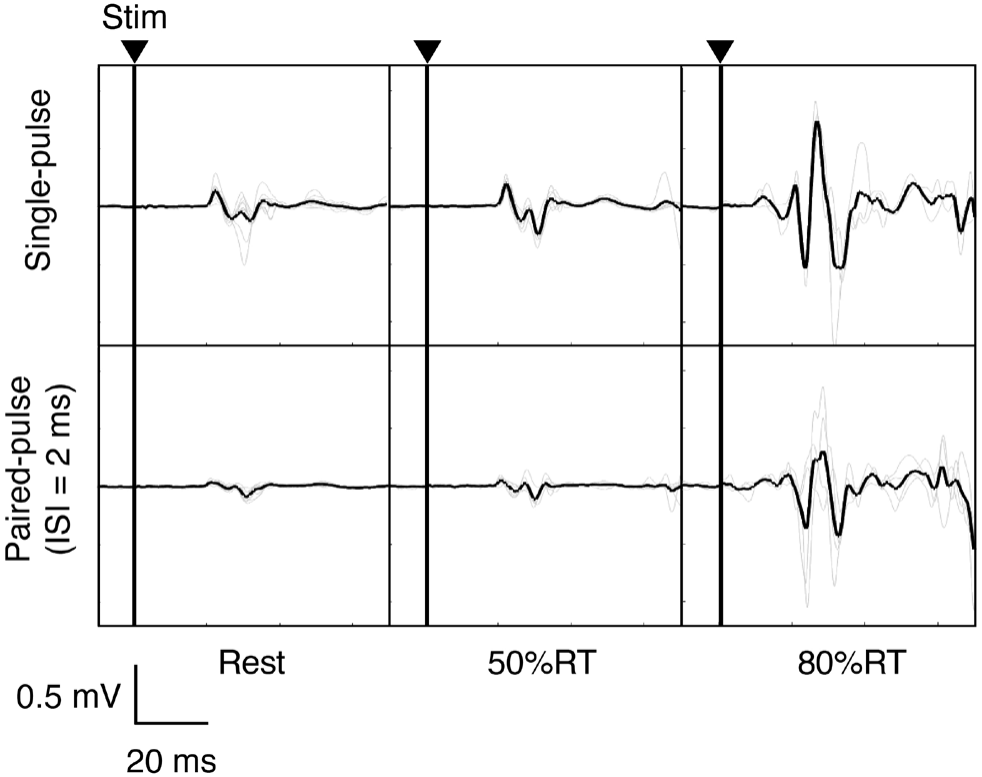
Representative signals of motor-evoked potentials (MEPs). Filtered electromyogram (EMG) signals from the right extensor digitorum communis (EDC) muscles during the TMS experiment. The upper and lower panels indicate MEPs from single-pulse and paired-pulse conditions, respectively.

To evaluate changes in corticospinal excitability and intracortical inhibition during movement preparation, we applied single- and paired-pulse TMS in the resting and pre-movement period to assess the changes of MEP and SICI (Fig. 5). MEP amplitude derived from EDC muscles at resting-state and movement preparation states from one participant indicated that motor preparation induces stronger MEP (Fig. 5). Moreover, SICI magnitude quantified by MEP amplitudes elicited by the paired-pulse TMS was also facilitated during movement preparation.

**Fig. 6.**
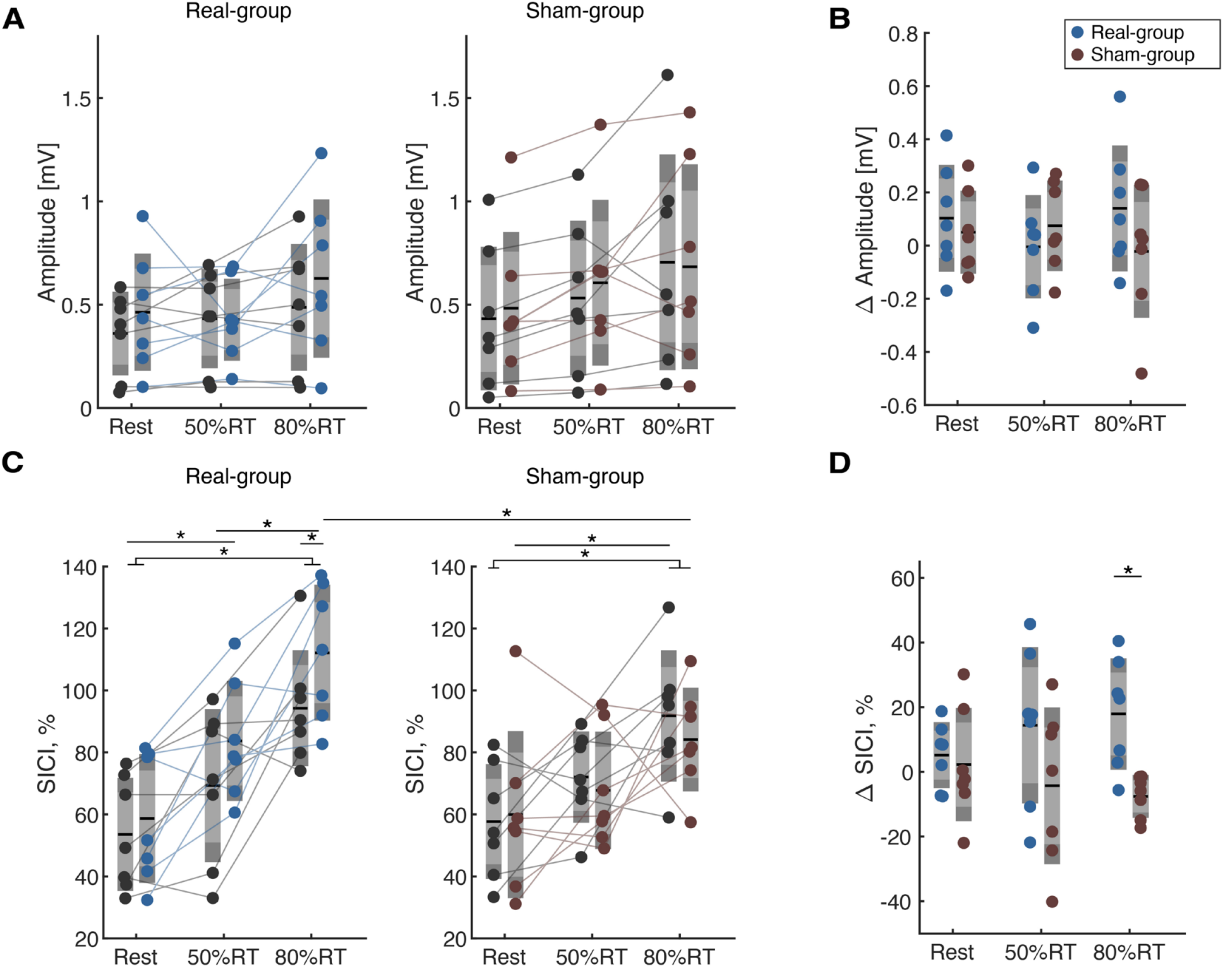
Single- and paired pulse stimulation during rest and pre-movement period**.**(A) MEP amplitude derived by single-pulse TMS. (B) Between-group comparison of changes in MEP. (C) Short-latency intracortical inhibition (SICI) magnitude derived by paired-pulse TMS. The relative MEP magnitude from single-pulse conditions is shown. A three-way repeated-measures ANOVA revealed significant interaction of Condition, Time, and Group effects (*p< 0.05). (D) Between-group comparison of changes in SICI magnitude. The post-hoc analysis revealed a significant difference in SICI at 80% RT (*p< 0.05).

The group-level analysis for single-pulse MEP amplitude using a three-way rmANOVA with Condition, Time, and Group factors indicated a significant main effect of Condition (*F*= 7.30,*p*= 0.011: Greenhouse-Geisser sphericity corrections,Fig. 6A). Post-hoc analysis revealed that MEP amplitude was significantly different between resting and 50%RT (*t*= -2.31,*p*= 0.029,*d*= 0.44), resting and 80%RT (*t*= -3.60,*p*= 0.001,*d*= 0.68), and 50% and 80%RT (*t*= -2.77,*p*= 0.01,*d*= 0.52). This indicates pre-movement corticospinal excitability was enhanced as the movement cue was provided. We tested changes in MEP amplitude using a two-way rmANOVA with Condition and Group factors (Fig. 6B). It revealed that MEP amplitude did not indicate a significant interaction (*F*= 1.31,*p*= 0.28), and main effects of Condition or Group (Condition:*F*= 0.16,*p*= 0.86; Group:*F*= 0.48,*p*= 0.50).

We tested SICI modulation using the three-way rmANOVA model (Fig. 6C). It indicated there was a significant interaction of Condition × Time × Group effect (*F*= 3.83,*p*= 0.041). Post-hoc analysis revealed that SICI significantly reduced during movement preparation (Rest vs 80%RT in both groups, Rest and 50%RT in the real group, all*p*< 0.05 with Bonferroni correction). In addition, the SICI reduction was significantly stronger at post-training evaluation in the real group at 80%RT (*t*= -4.602,*p*= 0.026,*d*= 1.52) and was also significantly decreased compared to the sham group (*t*= 4.695,*p*= 0.002,*d*= 2.55). We tested changes in SICI using a two-way rmANOVA with Condition and Group factors (Fig. 6D). It revealed that there was a significant interaction of Condition and Group effect (*F*= 3.83,*p*= 0.041), as well as main effects of Condition and Group (Condition:*F*= 6.81,*p*= 0.006; Group:*F*= 16.0,*p*= 0.003). The post-hoc analysis revealed that SICI was significantly reduced at 80%RT condition (*t*= 4.12,*p*= 0.005,*d*= 2.24).

### 3.4 Training-induced changes in sensorimotor rhythm voluntary controllability were associated with enhanced pre-movement downregulation of intracortical inhibition

**Fig. 7.**
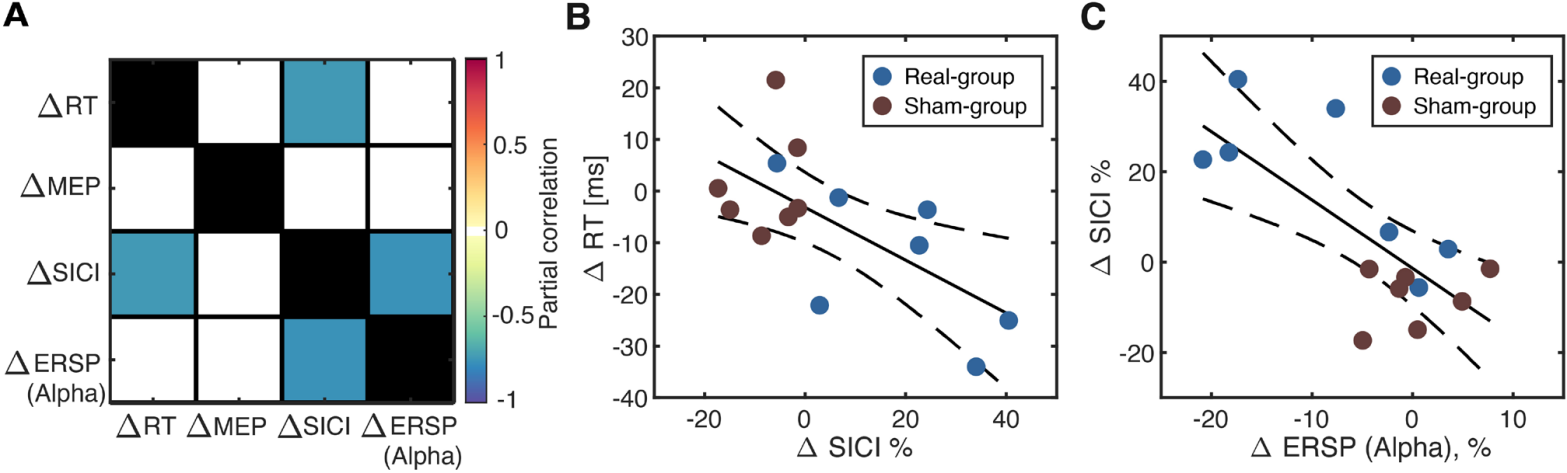
Correlation analysis for neurofeedback training-induced behavioral and neurophysiological changes. (A) Partial correlation analysis for the multimodal changes induced by NFT. All differences were computed as the difference from pre-evaluation data. Statistically significant pairs were colored. (B) Relationship between SICI and RT changes. The stronger downregulation of pre-movement SICI (Positive ∆SICI) was associated with the reduction of RT (Negative ∆RT), resulting in a significant negative correlation. (C) Relationship between ERSP and pre-movement SICI changes. The stronger SMR-ERD (Negative ∆ERSP) was associated with the stronger downregulation of pre-movement SICI (Positive ∆SICI), resulting in a significant negative correlation.

To explore the association among neurophysiological and psychophysical outcome measures such as EEG-SMR, MEP amplitude, pre-movement SICI magnitude, and reaction time, we explored the multivariate correlational structures (Fig. 7A). All participants from both groups were considered together. The partial correlation analysis revealed that modulations in SICI at 80%RT were negatively correlated with reaction time (*r*= -0.653,*p*= 0.01;Fig. 7B) and the modulations in ERSP at IAF (*r*= -0.738,*p*= 0.003;Fig. 7C) after regressing out the correlation among other variables.

## 4 Discussion

In the present study, we aimed to characterize the neurophysiological effects of NFT using SMR-ERD as a monitoring biomarker of SMC activities. To this end, we performed a multimodal assay including, EEG, TMS, and behavioral methods. The voluntary controllability of SMR-ERD and pre-movement downregulation of SICI were facilitated after the real NFT. These physiological changes were accompanied by a significant reduction in RT.

The behavioral improvement induced by NFT was quantified by a simple reaction time task, an established psychophysical task used in previous NFT studies (Chiew et al., 2012;Doppelmayr & Weber, 2011;He et al., 2020). This task is suitable for probing the relationship between NFT-induced changes in SMC activities and behavioral performance since the visually cued movement is generated at M1 without complex cortical computation, unlike tasks requiring variable responses depending on stimuli (e.g., go/nogo or serial reaction time task) (Aron et al., 2014;Kornysheva et al., 2019;Neubert et al., 2010). This means the RT of the simple reaction task can be significantly influenced by the excitability of the descending corticospinal tract where motor commands are conveyed, and the manipulated EEG-SMR-ERD is accompanied by M1 excitability modulation (Kraus et al., 2016,^2018^;Ros et al., 2010). Therefore, the task structure allowed us to capture behavioral improvement guided by short-term NFT for SMR-ERD regulation.

The behavioral improvement found in the NFT group suggests that motor imagery exercise combined with closed-loop neurofeedback can recruit the functionally overlapped neural population in M1 involved during real movement (i.e., simple reaction time task) (Churchland & Shenoy, 2024;Dekleva et al., 2024;Losey et al., 2024). The authors posit that the stronger SMR-ERD induced via NFT was associated with voluntarily induced M1 excitability change, and therefore, the combination of NFT and motor imagery lead to RT reduction. Meanwhile, we did not find the evidence of behavioral improvement for participants in the sham group. The yoked-sham placebo neurofeedback, which was inconsistent with their ongoing brain state, might have led to ineffective manipulation of the cortical excitability compared to the real group (Kober et al., 2018;Kodama et al., 2023;Soekadar et al., 2015). Collectively, the combination of NFT and motor imagery has systematically manipulated SMC excitability, contributing to the RT reduction.

The task-related activation of local neural circuits in M1 was assessed by a TMS experiment combined with the motor task (Coxon et al., 2006;Davey et al., 1998;Duclos et al., 2008;Duque & Ivry, 2009;Heise et al., 2013;Hummel et al., 2009). Specifically, we found a significantly stronger downregulation of SICI in the pre-movement period. As the paired-pulse protocol for SICI reflects the inhibitory activity mediated by the GABA_A_receptor in M1 (Kujirai et al., 1993), pre-stimulus SMR-ERD magnitude is associated with SICI downregulation (Takemi et al., 2013). Based on these correlational studies, the results of our manipulative approach suggest that SMR-ERD NFT reduced inhibitory activity in the motor cortex, leading to changes in cortical inhibitory circuits and potentially decreased RT.

The exploratory correlation analysis for the changes in the multi-layer neurophysiological metrics revealed a hierarchical relationship among scalp EEG representation of SMC activity (i.e., SMR-ERD), single-pulse and paired-pulse TMS, and behavioral performance (i.e., RT). A significantly negative correlation between changes in RT and SICI magnitude indicates that the disinhibition of SICI is associated with the reduction of RT, suggesting that the NFT manipulates the responsiveness of local M1 circuits as designed. Moreover, another significant correlation between SMR-ERD and SICI magnitude is consistent with the observation because the stronger SMR-ERD (negative value of ERSP) was associated with stronger SICI downregulation (positive SICI magnitude), suggesting that NFT-induced SMR-ERD leads to SICI downregulation in the pre-movement period, and therefore reduced RT.

**Fig. 8.**
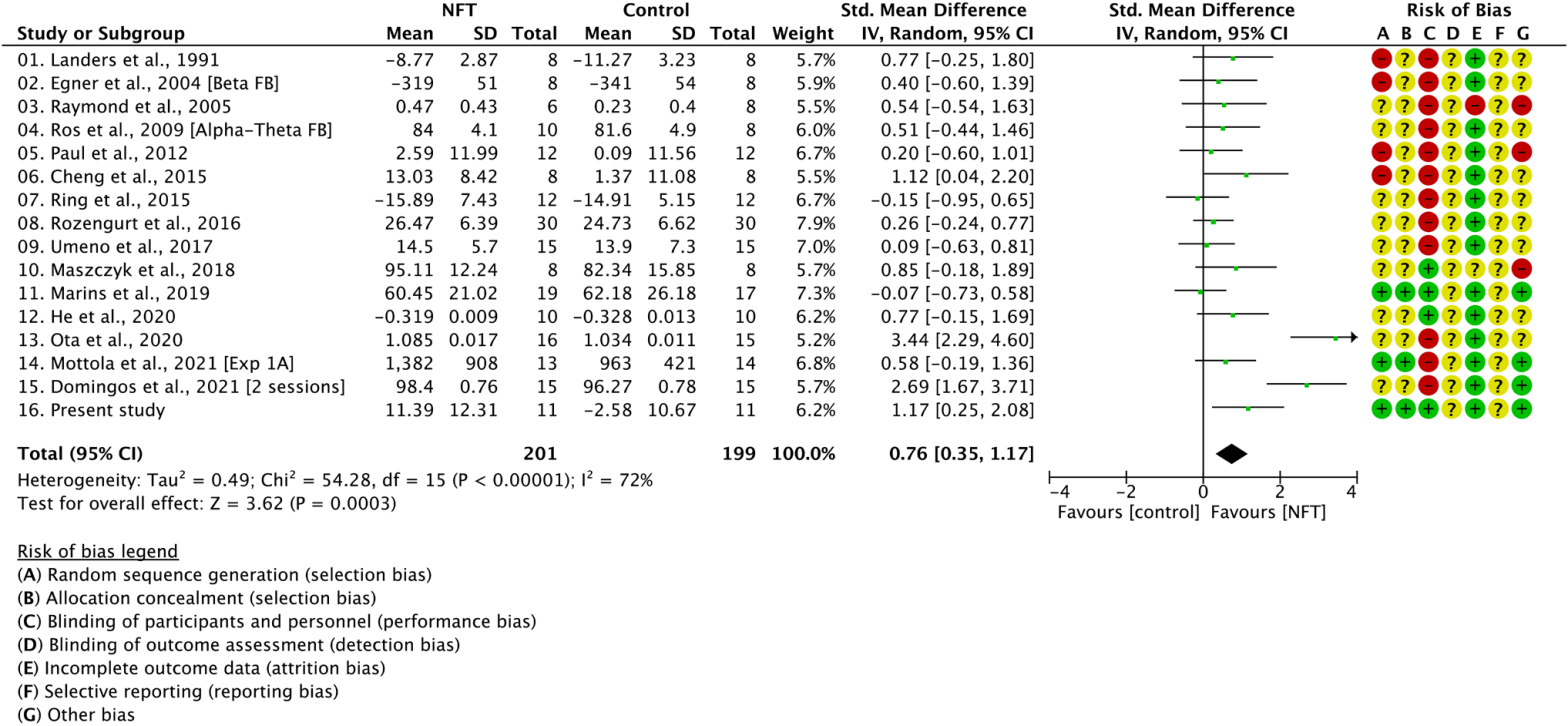
Forest plot and risk of bias for the efficacy of NFT in motor performances. The panel was adapted from our previous meta-analysis result (Onagawa et al., 2023) and modified to include the result of the present study. The left plot depicts overall effect size estimates (SMD) and a 95% CI. Risk of bias was assessed as green, low risk of bias; red, high risk of bias; yellow, unclear risk of bias.

Previous neurofeedback studies have demonstrated that the effect size of NFT in motor performance correlates with the intervention period (Onagawa et al., 2023). However, our NFT showed a large effect size compared to other studies even after a single-day training (Fig. 8). We posit that the reduction of the intervention period was achieved by our neurofeedback system designed to improve the quality of feedback signals. Specifically, in this study, we used Laplacian filters and IAFs to improve signal quality and the focality using the responsive spatio-spectral features (Pichiorri et al., 2015). Furthermore, visual feedback has also been reported to be more effective with feedback using hand images than with regular bar feedback (Ono et al., 2013). Collectively, we believe that these methods employed in our NFT contributed to the improvement in motor performance even after a single day of intervention.

The risk-of-bias assessment revealed that our study was designed to minimize the risk of bias, stemming from the insufficient description of randomization or allocation (Fig. 8). Moreover, based on the consensus on the reporting and experimental design of clinical and cognitive-behavioral neurofeedback studies (CRED-nf checklist:Ros et al., 2020), our study was conducted in accordance with the recent standard requirements for NFT studies. Although the present study was not pre-registered and thus we cannot exclude the risk of biased outcome assessment and selective reporting, we posit that our study provides results sufficiently reliable to conclude that the NFT-induced neural signaling changes were the main contributing factor to SMC reorganization to enhance pre-movement disinhibition.

The evidence is also expected to enhance the effectiveness of BCI rehabilitation (Cervera et al., 2018;Khademi et al., 2022;Ramos-Murguialday et al., 2013). In stroke patients, it has been reported that modulation of SICI during pre-movement is defective (Hummel et al., 2009). A current systematic review showed that sensorimotor NFT is effective in the rehabilitation of chronic stroke (Cervera et al., 2018;Nojima et al., 2021). These findings suggest that the mechanism of NFT for stroke rehabilitation may attenuate GABA_A_-mediated inhibition.

There are several limitations in this study that need to be considered when interpreting the results. First, we did not record EEG signals during pre- and post-behavioral tests. Therefore, we could not perform single-trial level analysis, such as beta burst or coherence analysis (Little et al., 2019). Since reaction time is related to beta bursts (Hussain et al., 2022;Khanna & Carmena, 2017;Little et al., 2019), it is necessary to examine EEG changes during movement preparation by NFT in the future. Additionally, the age range of participants was consequently in the late teens and 20s, as they were recruited from university. There was no experimental plan to generalize the effect of age. Because there were no older participants in the study, it is unclear whether our findings can be generalized to older people.

## Data Availability

The data that support the findings of this study are available from the corresponding author upon reasonable request. The source code is available fromhttps://github.com/Junichi-Ushiba-Laboratory/neurofeedback-induced-desynchronization-of-sensorimotor-rhythm
